# Shift of Choline/Betaine Pathway in Recombinant *Pseudomonas* for Cobalamin Biosynthesis and Abiotic Stress Protection

**DOI:** 10.3390/ijms232213934

**Published:** 2022-11-11

**Authors:** Larissa Balabanova, Iuliia Pentekhina, Olga Nedashkovskaya, Anton Degtyarenko, Valeria Grigorchuk, Yulia Yugay, Elena Vasyutkina, Olesya Kudinova, Aleksandra Seitkalieva, Lubov Slepchenko, Oksana Son, Liudmila Tekutyeva, Yury Shkryl

**Affiliations:** 1 Advanced Engineering School, Institute of Biotechnology, Bioengineering and Food Systems, Far Eastern Federal University, 10 Ajax Bay, Russky Island, 690922 Vladivostok, Russia; 2 Laboratory of Marine Biochemistry, G.B. Elyakov Pacific Institute of Bioorganic Chemistry, Far Eastern Branch, Russian Academy of Sciences, Prospect 100-letya Vladivostoka 152, 690022 Vladivostok, Russia; 3 ARNIKA, Territory of PDA Nadezhdinskaya, Centralnaya St. 42, Volno-Nadezhdinskoye, Primorsky Krai, 692481 Vladivostok, Russia; 4 Federal Scientific Center of the East Asia Terrestrial Biodiversity, Far East Branch, Russian Academy of Sciences, 159 Stoletija Str., 690022 Vladivostok, Russia

**Keywords:** *Pseudomonas denitrificans*, vitamin B12, *N*-methyltransferases, heterologous expression, chimeric enzymes, abiotic stress

## Abstract

The B12-producing strains *Pseudomonas nitroreducens* DSM 1650 and *Pseudomonas* sp. CCUG 2519 (both formerly *Pseudomonas denitrificans*), with the most distributed pathway among bacteria for exogenous choline/betaine utilization, are promising recombinant hosts for the endogenous production of B12 precursor betaine by direct methylation of bioavailable glycine or non-proteinogenic *β*-alanine. Two plasmid-based de novo betaine pathways, distinguished by their enzymes, have provided an expression of the genes encoding for *N*-methyltransferases of the halotolerant cyanobacterium *Aphanothece halophytica* or plant *Limonium latifolium* to synthesize the internal glycine betaine or *β*-alanine betaine, respectively. These betaines equally allowed the recombinant pseudomonads to grow effectively and to synthesize a high level of cobalamin, as well as to increase their protective properties against abiotic stresses to a degree comparable with the supplementation of an exogenous betaine. Both de novo betaine pathways significantly enforced the protection of bacterial cells against lowering temperature to 15 °C and increasing salinity to 400 mM of NaCl. However, the expression of the single plant-derived gene for the *β*-alanine-specific *N*-methyltransferase additionally increased the effectiveness of exogenous glycine betaine almost twofold on cobalamin biosynthesis, probably due to the *Pseudomonas*’ ability to use two independent pathways, their own choline/betaine pathway and the plant *β*-alanine betaine biosynthetic pathway.

## 1. Introduction

An oxidative derivate of choline, known as betaine, is an indispensable methyl-group donor for central metabolism and vitamin B12 biosynthesis, as well as an osmoprotectant and stabilizer of the protein quaternary structures in response to abiotic stresses. Betaine can either be consumed exogenously through nutrition intake or synthesized endogenously through a common biosynthesis pathway from choline, utilizing a two-step reaction conserved for bacteria and plants, but distinguished by the enzymes involved [1,2]. The choline utilization in Gram-negative bacteria, such as *Escherichia coli*, *Sinorhizobium meliloti*, and *Pseudomonas* spp., is carried out through the osmotically activated transporting systems with the high and low affinity BetT and ProU, followed by the oxidation of choline firstly to glycine betaine aldehyde by the choline dehydrogenase BetA. The second stage of oxidation, catalyzed by the glycine betaine aldehyde dehydrogenase BetB, leads to conversion of glycine betaine aldehyde into glycine betaine [1]. However, choline as the substrate for glycine betaine biosynthesis is a limiting factor due to its deficiency in nature and the cost of large-scale fermentation. Thus, the human and animal gut-associated bacteria under anaerobic conditions compete with the hosts for choline or significantly limit its bioavailability, inducing the metabolic diseases related to cardiovascular pathologies, and reducing the global DNA methylation [3,4,5]. Changes in DNA methylation have been found to associate closely with such human pathologies as cancer, aging, atherosclerosis, cognitive impairments including depression, anxiety, schizophrenia, or Alzheimer’s disease, and autoimmune disorders. The gut-bacterial choline metabolism converts a portion of choline into trimethylamine (TMA) and acetaldehyde under anaerobic conditions via the recently identified choline utilization (cut) gene cluster, which may cause choline and particularly B12 deficiencies either in a host or the B12-dependent strains [3,6,7]. Remarkably, the environmental *Pseudomonas*-like bacteria also have to enhance the choline/betaine uptake and the consequent oxygen-dependent B12 biosynthesis to supply it in the deeper layer of their cells for the strongly B12-dependent anaerobic growth in the biofilm [8]. Nevertheless, the decrease in choline uptake and TMA production (~100-fold for animals) by the choline-utilizing bacteria is feasible by the supplementation of betaine instead of choline through a specific diet [9,10].

The metabolism capability under betaine as the methyl-group donor during the industrial vitamin B12 production in *Pseudomonas denitrificans* was also significantly higher than that of choline chloride, with the improvement of B12 yield by the limiting of oxygen uptake [11,12,13]. The strategy of betaine control for vitamin B12 fermentation by *P. denitrificans* has shown an increased formation of some key precursors and intermediates facilitating vitamin B12 biosynthesis, such as δ-aminolevulinic acid (ALA, the first precursor of vitamin B12), glutamate (an intermediate of ALA via the C5 pathway), glycine (an intermediate of ALA via the C4 pathway), and methionine (directly participating in the methylation reaction involved in the vitamin B12 biosynthetic pathway) [12,13]. However, the most effective and economical strategy of betaine feeding, established for vitamin B12 fermentation, was continuously maintaining the betaine concentration of the broth at the range of 5–7 g/L during 50–140 h of fermentation [14].

Recently, an alternative pathway for internal betaine synthesis from more readily available amino acid glycine has been described in halotolerant cyanobacteria [1,15]. The pathway was discovered in *Aphanothece halophytica* that consists of three-stage sequential reactions of glycine methylation mediated by a pair of *N*-methyltransferases. The *A. halophytica* glycinsarcosine methyltransferase ApGSMT catalyzes methylation of glycine to sarcosine and sarcosine to dimethylglycine, respectively, and the *A. halophytica* dimethylglycine methyltransferase ApDMT catalyzes the specific methylation of dimethylglycine to betaine [15].

Whereas an engineering of the *A. halophytica* glycine betaine (GB) synthesis requires installing two genes for glycine methylation, the production of another known osmoprotectant β-alanine betaine (*β*AB) could be provided by only expressing a single gene encoding for the *N*-methyltransferase, which is specific to the non-protein *β*-alanine in a stress-tolerant plant, *Limonium latifolium*, a member of the halophytic family Plumbaginaceae [16]. The *β*AB synthesis is not constrained by choline and oxygen availability; therefore, it is suggested to be suitable for osmoprotection under saline and hypoxic conditions [16].

In this study, we obtained the recombinant betaine producers from the strains *Pseudomonas* sp. CCUG 2519 and *P. nitroreducens* DSM 1650 (both formerly *P. denitrificans*), modified by the different genetic constructs containing the *N*-methyltransferases genes of the cyanobacterium *A. halophytica* and the plant *L. latifolium*, to compare the effects of choline/betaine pathways on the level of cobalamin synthesis and abiotic stress protection.

## 2. Results and Discussion

### 2.1. The Effect of Recombinant N-Methyltransferases Expression on Bacterial Growth Rate under Stress Conditions

**Figure 1:**
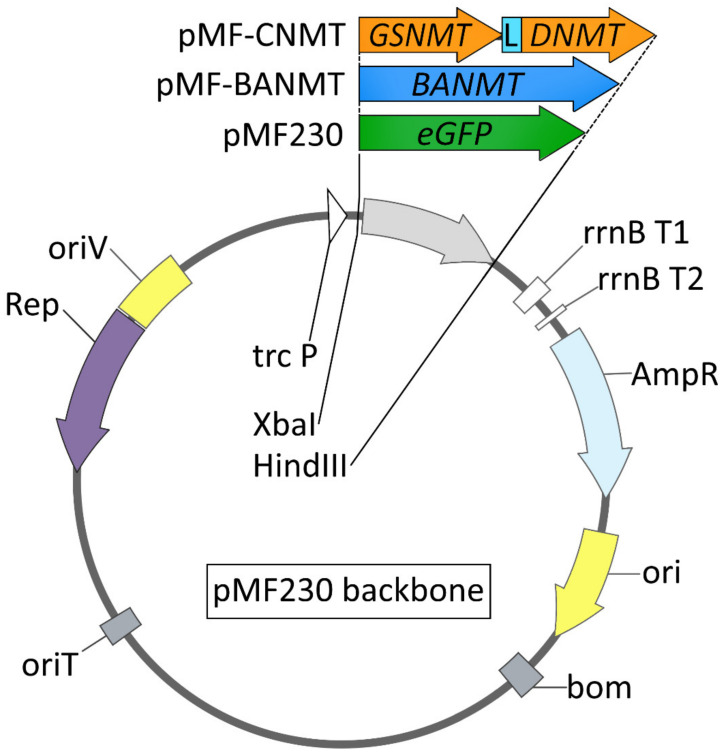
Schematic representation of plasmid vectors used in this study: trc P, hybrid promoter; lac, lac operator; rrnB T1, rrnB T2, transcription terminators from *rrnB* gene; AmpR P, *β*-lactamase gene promoter; AmpR, *β*-lactamase gene; ori, ColE1 origin of replication; bom, basis of mobility region from pBR322; oriT, incP origin of transfer; oriV, broad-host-range origin of replication from *Pseudomonas aeruginosa* plasmid pRO1600; Rep, replication protein gene for the pRO1600 plasmid; *eGFP*, enhanced green fluorescent protein; *BANMT*, *β*-alanine *N*-methyltransferase gene from *Limonium latifolium*; *GSNMT*, glycine sarcosine *N*-methyltransferase gene from *Aphanothece halophytica*; *DNMT*, dimethylglycine *N*-methyltransferase gene from *A. halophytica*; L, the sequence, encoding the Gly-Gly-Gly-Gly-Se [(GGGGS)x3] linker peptide (the fusion of *GSNMT*, *L*, and *DNMT* creates an in-frame chimeric *N*-methyltransferase gene, *CNMT*).

The recombinant *Pseudomonas* sp. CCUG 2519 and *P. nitroreducens* DSM 1650 strains carrying the pMF-BANMT or pMF-CNMT expression genetic constructs were obtained using the 3-parental mating method as described in Figure 1. The identity of the obtained strains was confirmed by the resistance to the corresponding selection markers and by the PCR-based method. The new strains *Pseudomonas* sp. CCUG 2519/pMF-BANMT and *P. nitroreducens* DSM 1650/pMF-BANMT, as well as *Pseudomonas* sp. CCUG 2519/pMF-CNMT and *P. nitroreducens* DSM 1650/pMF-CNMT, are capable of producing betaines, namely: *β*-alanine betaine (*β*AB) and glycine betaine (GB), respectively, which were confirmed by tandem mass spectrometry analysis (Supplementary Figure S1).

**Figure 2:**
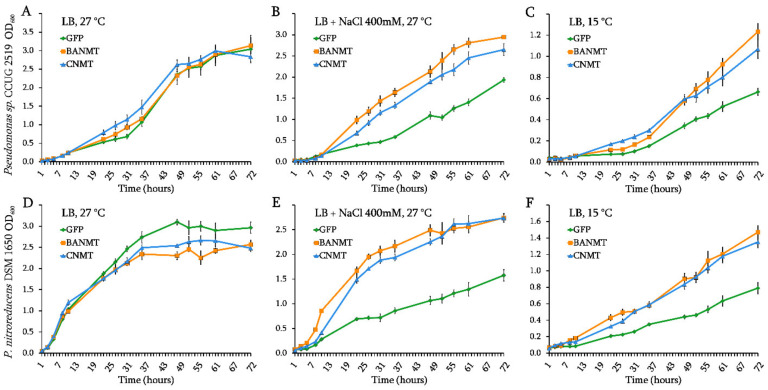
The effect of *eGFP*, *CNMT* and *LlBANMT* gene expression on the growth dynamics of *Pseudomonas* sp. CCUG2519 (**A**–**C**) and *P. nitroreducens* DSM1650 (**D**–**F**) strains under the control conditions (LB, 27 °C), high salinity (LB + 400mM NaCl), and cold stress (LB, 15 °C). Data are the means ± standard errors of the mean of three independent biological experiments with three technical replicates each.

To investigate the effect of the recombinant *N*-methyltransferase gene expression on the bacterial growth dynamics and their ability to withstand abiotic stresses, the recombinant strains of *Pseudomonas* sp. CCUG 2519 and *P. nitroreducens* DSM 1650 expressing either the control fluorescent *eGFP* or the plant *LlBANMT* and bacterial *CNMT* genes were tested. Under the normal culture conditions, the presence of recombinant methyltransferase genes did not significantly affect the bacterial growth rate. The recombinant strains reached their maximum optical density under the given cultivation conditions simultaneously (Figure 2A,D). It was previously shown that the expression of *GSNMT* and *DNMT* genes, derived from the halotolerant bacterium *A. halophytica*, significantly increased the threshold salt concentration in the medium at which the recombinant *Synechococcus* sp. PCC 7942 cells are able to grow [15]. The introduction of a genetic construct that ensures both activities of the *GSNMT* and *DNMT* genes led the bacterial cells to lower their sensitivity to an increased salinity [15]. However, it was unclear whether the pseudomonads would have similar characteristics. In addition, in our experiments, these two genes were expressed in the form of chimeric protein *CNMT* (Figure 1).

Actually, the growth rate of the *eGFP*-expressing strains, which were used as the control, slowed down significantly in the presence of 400 mM NaCl, preventing the bacterial cultures from reaching their normal optical density values even after 72 h of cultivation (Figure 2B,E). However, no significant decrease in the growth rate was observed in the recombinant strains of both *Pseudomonas* species expressing the chimeric *CNMT* gene, suggesting that its chimeric product fulfilled the function of the entire enzymatic pathway (Figure 2B,E).

Whereas the effect of *β*AB biosynthesis gene expression on the resistance to salt stress has yet to be elucidated, there are a number of studies demonstrating the effectiveness of exogenous GB as a factor of the bacterial resistance to a high salinity [16,17,18,19]. Due to the similarity of physical and chemical properties of GB and *β*AB, it can be assumed that the capability of endogenous *β*AB accumulation will also help to reduce the effect of salt stress on the transgenic cells. In our experiment, no growth retardation was observed on the medium containing 400 mM NaCl for both *Pseudomonas* species expressing the *LlBANMT* gene, which confirms the hypothesis of the protective effect of the plant gene in relation to the increased environmental salinity (Figure 2B,E).

Some bacteria have been found to activate the GB accumulation pathways (which include synthesis and uptake) as an adaptation to low temperatures [20], with even low concentrations of betaine found to be sufficient to protect bacteria from cold stress [21]. Thus, it was interesting to test whether the introduction of new betaine biosynthesis genes could affect the ability of *Pseudomonas* to withstand low temperatures. A significant slowdown in the growth rate was observed in both wild-type *Pseudomonas* species when the cultivation temperature was reduced to 15 °C (Figure 2C,F).

However, the presence of either vector containing the betaine biosynthesis pathway genes reduced the negative effect of low temperature on the growth of the recombinant strains of *Pseudomonas* sp. CCUG 2519 and *P. nitroreducens* DSM 1650, compared to their control *eGFP*-expressing strains (Figure 2C,F). This suggests that the accumulated amounts of both endogenous glycine betaine and *β*-alanine betaine are equally sufficient for cryoprotective activity (Figure 2C,F).

### 2.2. The Effect of Recombinant N-Methyltransferases Expression on Vitamin B12 Production

**Figure 3:**
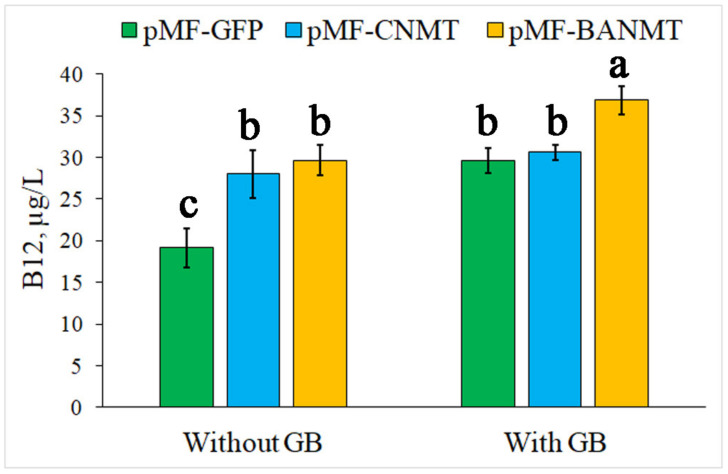
The effect of *eGFP*, *CNMT*, and *LlBANMT* genes’ expression on vitamin B12 production in the recombinant *P. nitroreducens* DSM 1650 strains. Data are the means ± standard errors of the mean of three independent biological experiments with three technical replicates each. Different letters above the bars indicate statistically significant differences in means (*p* < 0.05), Fisher’s LSD.

The results on B12 productivity in the cells of *P. nitroreducens* DSM 1650 (H.G. Schlegel (*P. denitrificans*) = ATCC 13867), estimated by the B12 concentration, show that the mutant strains carrying both *CNMT* and *LlBANMT* genes have become less dependent on the exogenous GB for B12 synthesis than the wild-type strain (Figure 3). The difference between the B12 productivity in the wild-type strain grown with and without GB is more significant than in the *CNMT* and *LlBANMT* mutants. In general, the B12 productivity in the mutants, particularly in the *LlBANMT*-containing strain, is also enhanced in comparison with the wild-type strain at the supplementation of exogenous GB (Figure 3).

It is evident that the addition of betaine in the nutrition medium significantly (65%) increases the yield of B12 in the control cells. Recently, the mechanism of the promoting effect of betaine as a methyl-group donor for vitamin B12 biosynthesis has been concluded from a proteomic analysis of *P. denitrificans* [22]. At the same time, the B12 accumulation level in the *CNMT*-expressing strain approximately corresponds to that value in the wild-type strain grown with GB (Figure 3). However, the additional GB in the media was not able to further stimulate B12 biosynthesis in this strain (Figure 3). Similarly, the content of B12 in the *LlBANMT*-expressing bacteria corresponds to the control *P. nitroreducens* DSM 1650 culture supplemented with GB, as well as to the pMF-CNMT transformed cells both with and without the additional GB. In contrast to the *CNMT*-expressing bacteria, the exogenous GB stimulated the accumulation of B12 by 80% in the pMF-BANMT transformed cells, which approximately corresponds to the native activity of GB in relation to the vitamin B12 biosynthesis pathway in the control *P. nitroreducens* DSM 1650 cells (Figure 3).

Thus, the recombinant B12-producing *P. nitroreducens* strain containing the fusion of *GSNMT* and *DNMT* genes from halotolerant bacteria expresses the chimeric enzyme with the cooperative *N*-methyltransferase activity for the biosynthesis of glycine betaine from glycine, bypassing the need to use the native choline/betaine uptake and synthesis pathway [1,12,14,15]. An increase in the intracellular concentration of glycine betaine in the recombinant *CNMT*-expressing strain is probably equivalent to the inducing strength of the exogenous GB supplementation (Figure 3).

The *LlBANMT*-carrying strain of *P. nitroreducens* expresses the enzyme that converts alanine into another betaine derivative, *β*AB, whose potential turned out to be equal to the exogenous GB supplementation of the untransformed *P. nitroreducens* or de novo synthesized GB in the *CNMT*-expressing strain (Figure 3). However, the additional GB supplementation made it possible to use independent pathways for activating B12 biosynthesis in the *LlBANMT* mutant that increased the total vitamin B12 yield by a factor of two compared with the uninduced control and by about 25% compared with other strains in the presence of exogenous GB (Figure 3).

## 3. Materials and Methods

### 3.1. Bacterial Strains and Culture Conditions

The strains *Pseudomonas* sp. CCUG 2519 (formerly *Pseudomonas denitrificans* CCUG 2519) and *Pseudomonas nitroreducens* DSM 1650 (H.G. Schlegel (*Pseudomonas denitrificans*) = ATCC 13867) were purchased from the respective Collections of Microorganisms.

NGS sequencing (Illumina, Inc., San Diego, CA, USA) was carried out for the species identification and whole-genome analysis of the choline/betaine uptake and biosynthesis and cobalamin biosynthesis pathways as described earlier [23].

All chemicals denoted here were purchased from Sigma-Aldrich Corp. (St. Louis, MO, USA).

The strains were grown in Luria–Bertani medium (10 g peptone, 5 g yeast extract, and 10 g NaCl) at 30 °C for routine cultivation. For vitamin B12 production by the strains, three steps of cultivation were conducted. The first medium containing per liter: 30 g sucrose, 10 g peptone, 0.25 g (NH_4_)_2_SO_4_, 1.5 g (NH_4_)_2_HPO_4_, 0.1 g MnSO_4_ × H_2_O, 0.1 g ZnSO_4_ × 7 H_2_O, 20 g agar, with addition of 1.0 M NaOH (pH 7.2) before an autoclave, was used for the 24 h plate growth at 30 °C. Then, single colonies were inoculated into the second medium (15 mL) consisting of: (per liter) 30 g sucrose, 5 g KH_2_PO_4_, 2.3 g (NH4)_2_SO_4_, 0.7 g (NH_4_)_2_HPO_4_, 0.2 g MnSO_4_ × H_2_O, 1.5 g MgSO_4_, 0.02 g ZnSO_4_ × 7H_2_O, 0.02 g CoCl_2_ × 6H_2_O, 0.0045 g 5,6-dimethylbenzimidazole (DMBI), with addition of 1.0 M NaOH (pH 7.0–7.2) before autoclave, and grown for 48 h at 30 °C, 150 rpm. The cultured cells (ratio 1:100) were inoculated in 30 mL of the third medium (per liter): 20 g malt extract, 20 g glycine betaine, 1 g (NH_4_)_2_SO_4_, 1.5 g MgSO_4_, 0.75 g KH_2_PO_4_, 0.08 g ZnSO_4_ × 7H_2_O, 0.14 g CoCl_2_ × 6H_2_O, 0.075 g 5,6-DMBI, with addition of 1.0 M NaOH (pH 7.2–7.4) before autoclave, and grown for 5 d at 30 °C, 150 rpm. Control cells of each strain were cultured without glycine betaine. 

When the bacterial strains containing the pMF230 and pRK2013 plasmids were analyzed, 200 µg of ampicillin and 50 µg of kanamycin, respectively, per mL were added to the appropriate medium.

The strain *E. coli* DH5α was used for plasmid propagation.

### 3.2. Construction of Recombinant Plasmids and Gene Transfer by Conjugation 

For the expression of methyltransferase genes in the bacterial strains, the plasmid vectors were created using pMF230 as a backbone [24]. The plasmid pMF230 was purchased from Addgene (#62546; Watertown, MA, USA). This plasmid provides a broad host range replication origin and constitutive heterologous expression driven by a strong *trc-*based promoter, which is a hybrid of the *trp* and *lacUV5* promoters [25,26]. 

Seeds of *Limonium latifolium* were purchased from a local seed market and germinated under appropriate conditions. The total RNA was isolated from 4-week-old seedlings by using the LiCl method as described previously [27]. The first-strand cDNA synthesis was conducted with an RNAscribe RT kit (Biolabmix, Novosibirsk, Russia) according to the manufacturer’s instructions. To amplify the full-length coding sequence of the *L. latifolium β*-alanine-*N*-methyltransferase gene (*Ll*BANMT; Genbank Acc. No. AY216903), the polymerase chain reactions (PCRs) were carried out with the gene-specific primers: forward 5′CAG TCT AGA GTC GAC AGG AGA AGA AAA ATG GCG AAC CAC TCC TCA G3′ (*Xba*I restriction site is underlined) and reverse 5′ATG AAG CTT ACT TCT GGA ACT CTA CCA CGG3′ (*Hind*III restriction site is underlined). As soon as CDS of *Ll*BANMT contained internal *Xba*I and *Hind*III recognition sequences, the site-directed mutagenesis was further performed. The PCR reactions with the gene-specific primers, forward 5′GGC CAT TTC TTC ACA CAG CAA TCC TAG AC3′ and reverse 5′GCT GTG TGA AGA AAT GGC CAG GCT TTC3′, allowed us to eliminate *Xba* I and *Hind* III sites, without altering the deduced amino acid composition of *Ll*BANMT protein. The modified *Ll*BANMT coding sequence was sub-cloned as *Xba*I-*Hind*III fragment into the same sites of the linearized plasmids pMF230, whereby pMF-BANMT vector was obtained (Figure 3). The chimeric *N*-methyltransferase gene *CNMT* was designed as an in-frame fusion of the glycine sarcosine *N*-methyltransferase AhGSNMT (*GSNMT*; Genbank Acc. No. AB094497) and dimethylglycine *N*-methyltransferase AhDNMT (*DNMT*; Genbank Acc. No. AB094498) genes from the halotolerant cyanobacterium *A. halophytica* [15].

The coding sequences of AhGSNMT and AhDNMT were separated by a flexible peptide linker (GGGGS)x3. The synthesis of the *CNMT* gene was performed by Twist Bioscience (South San Francisco, CA, USA). The 1708-bp fragment of *CNMT* was obtained using the gene-specific primers: forward 5′TAT CTA GAG TCG ACA GGA GAA GAA AAA TGG CTA TCA AAG AAA AAC AAG 3′ (*Xba*I-restriction site is underlined) and reverse 5′TTA AGC TTC TAG GGT TTG TGG AAC TTG3′ (*Hind*III-restriction site is underlined). This fragment was sub-cloned as *Xba*I-*Hind*III fragment into the same sites of the linearized plasmids pMF230, whereby pMF-CNMT vector was obtained (Figure 1). Both the newly constructed plasmids, pMF-BANMT and pMF-CNMT, and the original pMF230 vector were mobilized into each strain of *Pseudomonas* sp. CCUG 2519 (formerly *P. denitrificans* CCUG 2519) and *P. nitroreducens* DSM 1650 (H.G. Schlegel (*P. denitrificans*) = ATCC 13867) by triparental mating using *E. coli* XL1 pRK2013 as a helper strain [28].

### 3.3. Mass Spectrometry-Based Identification of β-Alanine Betaine and Glycine Betaine

The recombinant *Pseudomonas* sp. CCUG 2519 and *P. nitroreducens* DSM 1650 strains harboring the pMF-BANMT or pMF-CNMT vectors were grown as described above, without an exogenous betaine supplementation. The cells were collected and washed using successive centrifugation at 4000 g. The pellets were then resuspended in a methanol/water (1:1) solution and incubated in an Elmasonic S15H ultrasonic bath (Elma Electronic, Wetzikon, Switzerland) for 1 h at a room temperature. The resulting extract was centrifuged at 17,000 g for 20 min. Finally, the supernatant was filtered through a GE Whatman 0.45 µm filter and used for the analysis.

Determination of *β*-alanine betaine and glycine betaine was carried out by tandem mass spectrometric detection with a Bruker HCT ultra PTM Discovery System (Bruker Daltonik, GmbH, Bremen, Germany). The MS signal was recorded the electrospray ionization mode and positive ion detection. The signal intensity of selected ions was monitored: [M+H]^+^ ions for β-alanine betaine, m/z 132; [M+H]^+^ and [2M+H]^+^ ions for glycine betaine, m/z 118 and m/z 235, respectively. The m/z scan range was 50–400; the desiccant gas flow (N_2_) was 8.0 L/min; the nebulizer gas pressure (N_2_) was 25.0 psi; the ion source potential was 4.0 kV; the dryer gas temperature was 320 °C. Tandem mass spectrometry was performed at a fragmentation voltage of 1 V.

The experiments described in this work were performed using equipment from the Instrumental Centre for Biotechnology and Gene Engineering at the Federal Scientific Centre of the East Asia Terrestrial Biodiversity of the Far East Branch of the Russian Academy of Sciences.

### 3.4. Determination of Bacterial Growth Rate under Abiotic Stress Conditions

The strains *Pseudomonas* sp. CCUG 2519 and *P. nitroreducens* DSM 1650, transformed with the pMF230, pMF-BANMT and pMF-CNMT plasmids, were stored in a 20% glycerol solution at −80 °C, from which they plated on LB agar containing 150 mg/L ampicillin and were incubated at 27 °C for three and two days, respectively. The individual colonies were tested for the presence of respective plasmids by PCR. The positive colonies were then inoculated into 20 mL LB containing 150 mg/L ampicillin (Ap) and grown overnight in an orbital shaker (SPH-200B, HEB, Shaanxi, China) at 27 °C, 250 rpm. The following day, the optical density of these cultures was measured and they were diluted in LB containing 100 mg/L Ap (or LB with Ap and 400mM NaCl for the high-salinity experiment) to reach the predesigned values of OD_600_ = 0.05. Falcon tubes (50 mL) each containing 15 mL of this initial culture were placed in rotary shakers at an angle of 30° from the horizontal at 27 °C, 170 rpm and 15 °C (for the cold stress experiment), 170 rpm. The optical density of bacterial cultures was monitored at 1, 3, 5, 8, 10, 22, 26, 35, 47, 51, 55, 60, and 72 h. Optical density at a wavelength of 600 nm (OD_600_) was analyzed on a Benchmark Plus Microplate Spectrophotometer System (Bio-Rad, Hercules, CA, USA) in 3 technical replicates with LB medium as the blank, using 200 µL/well in a 96-well plate. Three independent biological replicates of this experiment were performed and three technical replicates were analyzed for each biological replicate.

### 3.5. Measurement of Vitamin B12 Concentration

The enzyme immunoassay kit RIDASCREEN FAST Vitamin B12 (Art. No.: R2103; R-Biopharm AG, Darmstadt, Germany) was used for the quantitative determination of vitamin B12. For vitamin B12 production, the single colonies of both mutant and wild-type strains of *P. nitroreducens* DSM 1650 (H.G. Schlegel (*P. denitrificans*) = ATCC 13867) were cultivated in 30 mL of culture at the same conditions without exogenous betaine as described above in triplicate, then centrifuged at 4000 g for 5 min. The control colonies of the strains of *P. nitroreducens* DSM 1650 either containing recombinant plasmids or without them were cultivated with the exogenous glycine betaine. The supernatant was discarded, and 5 mL of Milli-Q water was added to the sediment. The precipitate was homogenized with an ultrasonic disintegrator (Q500 Sonicator, Qsonica) until the solution was transparent. One mL of the homogenate was transferred to a 1.5 mL Eppendorf tube and centrifuged at 23,000 g for 20 min. A total of 100 µL of supernatant was added to 100 µL of the dilution buffer and mixed thoroughly by pipetting. A total of 50 µL of this solution was taken for the analysis. The manufacturer’s instructions were followed. Three independent biological replicates of this experiment were performed, and three technical replicates were analyzed for each biological replicate.

### 3.6. Statistical Analysis

All values are expressed as the mean ± standard errors. For statistical evaluation, analysis of variance (ANOVA) followed by a multiple comparison procedure were employed using STATISTICA (Data Analysis Software System), Version 10. Fisher’s protected least significant difference (PLSD) post hoc test was employed for the inter-group comparison. The level of statistical significance was set at *p* < 0.05.

## 4. Conclusions

The *P. denitrificans-*like pseudomonads, capable of vitamin B12 production, can be effectively modified by the de novo betaine biosynthesis pathways on the base of plasmid pMF230 for shifting their traditional choline/betaine uptake pathway towards the biosynthesis of internal B12 precursors, GB or *β*AB. The recombinant strains could be provided by an endogenous betaine due to a heterologous expression either of two cyanobacterial or one plant-derived *N*-methyltransferases via enzymatic conversion of bioavailable glycine or non-proteinogenic *β*-alanine, respectively. Both de novo betaine pathways equally enforce the protection of bacterial cells against lowering temperature and increasing salinity that approximately correspond to the effect of exogenous glycine betaine, when their native pathway for choline/betaine transport and synthesis is stimulated. 

The plant *N*-methyltransferase *LlBANMT*, catalyzing conversion of the non-proteinogenic amino acid *β*-alanine into *β*AB, allows the recombinant pseudomonads to increase almost twofold the effectiveness of the GB supplementation on the B12 yield. Probably, the increase in the cobalamin level in the recombinant *Pseudomonas* is due to the use of two independent pathways for the production of precursor betaine to donate the additional methyl groups during the cobalamin synthesis pathway realization.

## Supplementary Materials

The following supporting information can be downloaded at: https://www.mdpi.com/article/10.3390/ijms232213934/s1.
