# Supplementary material for: Shift of Choline/Betaine Pathway in Recombinant Pseudomonas for Cobalamin Biosynthesis and Abiotic Stress Protection
Source: Int J Mol Sci. 2022 Nov 11;23(22):13934. doi: 10.3390/ijms232213934 (PMC9699165; doi:10.3390/ijms232213934)
Supplement: Supplementary file 1 [file ijms-23-13934-s001.zip › ijms-2003135-supplementary.pdf]

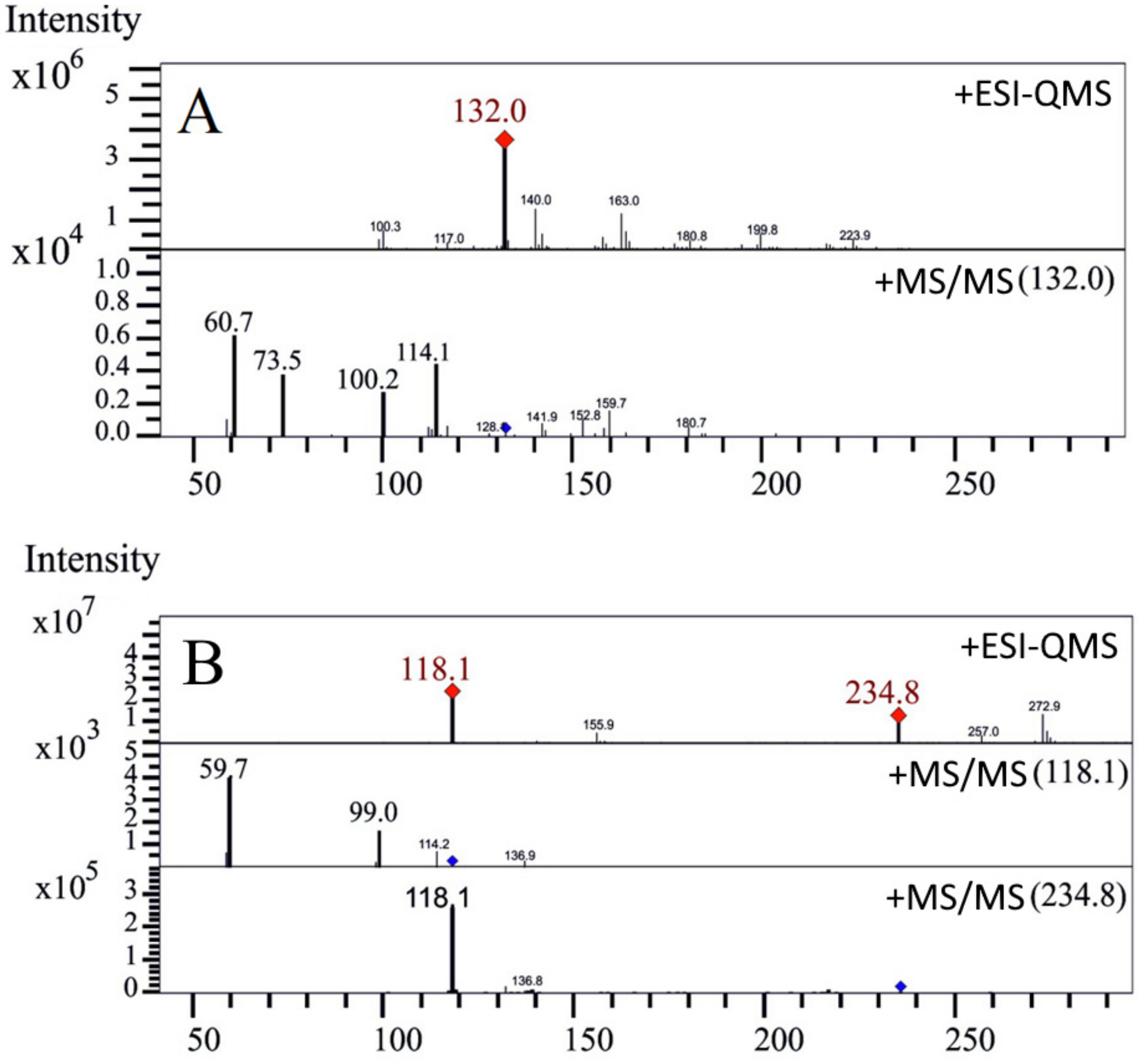

Supplementary Figure S1. The positive ESI-QMS and MS/MS spectra of betaine derivatives. (A) β-alanine betaine m/z 132 = $[M+H]^+$, m/z 114 = $[M\text{-}H_2O]$, m/z 100 = $[M\text{-}2CH_2]$, m/z 74 = $[M\text{-}3N(CH_3)_3]$, m/z 60 = $[M\text{-}CH_2CHCOOH]$, (B) glycine betaine m/z 235 = $[2M+H]^+$, m/z 118 = $[M+H]^+$, m/z 59 = $[M\text{-}CH_2COO]$. QMS – quadrupole mass spectrometry; MS/MS – tandem mass spectrometry.
